# Point-of-care ultrasound identification of pneumatosis intestinalis in pediatric abdominal pain: a case report

**DOI:** 10.1186/s13089-017-0057-0

**Published:** 2017-01-19

**Authors:** Vigil James, Aswin Warier, Khai Pin Lee, Gene Yong-Kwang Ong

**Affiliations:** Children’s Emergency, KK Women’s and Children’s Hospital, 100 Bukit Timah Road, Singapore, 229899 Singapore

## Abstract

We describe a case report of an infant with intussusception who presented to a pediatric emergency department with diarrhea and increased irritability. Pneumatosis intestinalis (intra-mural air) detected on point-of-care ultrasonography (but not apparent on plain abdominal radiographs) alerted the emergency physicians towards the severity of disease process.

## Background

The presence of intramural air in the bowel wall or pneumatosis intestinalis in pediatric population can be due to wide range of abdominal and non-abdominal conditions. Abdominal conditions include necrotizing enterocolitis, ileo-colic intussusception with bowel ischemia, venous occlusion of the bowel vasculature and inflammatory bowel disease [1–4]. Non-abdominal conditions that can result in pneumatosis intestinalis include asthma, bronchopulmonary dysplasia, post-bone marrow transplantation, congenital heart disease, juvenile rheumatoid arthritis, hemolytic anemia and generalized vascular hypo-perfusion [5, 6].

Children with acute intussusception present with severe abdominal pain to the emergency department (ED) [7]. For children who are appearing prostrated and sick with signs of bowel obstruction, urgent interventions towards diagnosis and management are vital to decrease morbidity and mortality. A point-of-care ultrasonography (POCUS) done by the emergency physician is shown to be associated with faster diagnosis time in a large variety of conditions [8–12] and use of POCUS to diagnose intra-abdominal pathologies is becoming more popular [13]. This case is the first description of the use of POCUS by pediatric emergency physicians to detect pneumatosis intestinalis in acute abdominal pain in children.

## Case

**Fig. 1:**
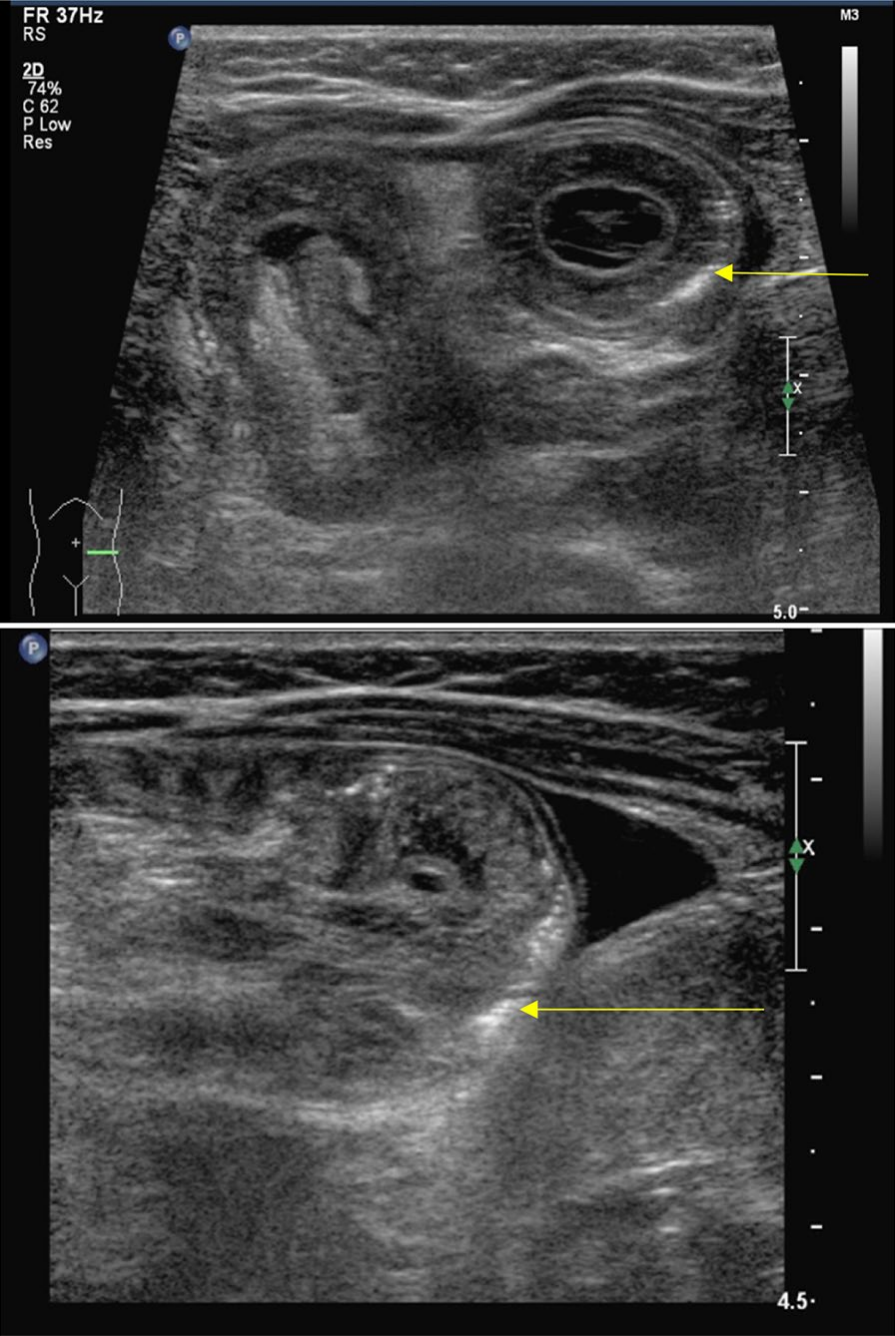
Abdominal POCUS (over *left upper quadrant*): showing intramural air (*yellow arrow*) in intussusception in a 3-month-old

**Fig. 2:**
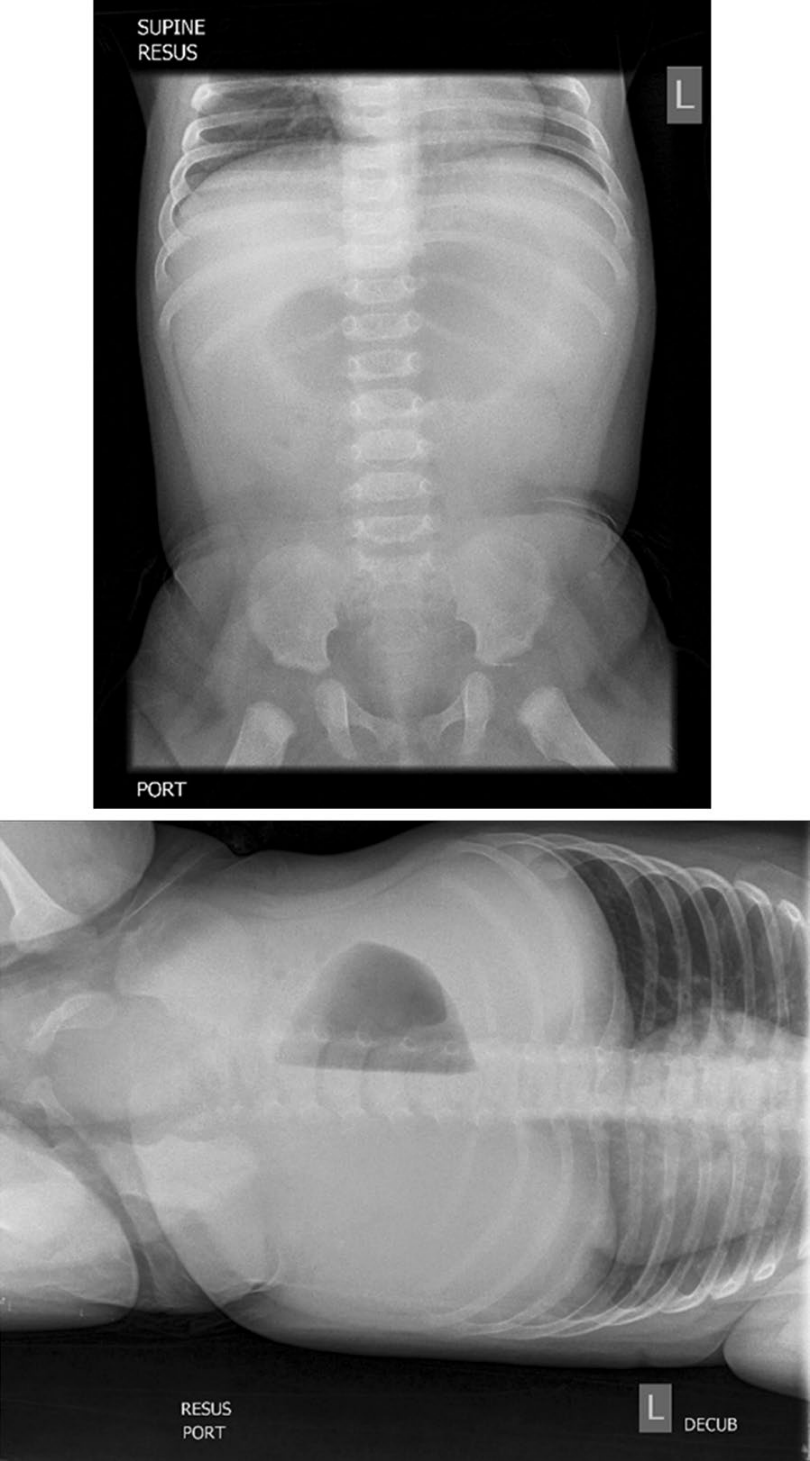
Abdominal X-rays: supine and left lateral decubitus radiographs showing a gasless abdominal field except in the gastric area. There were no obvious radiographic features of peumatosis or intussusception for the infant

A 3-month-old baby boy, who was previously well, presented with diarrhea for 1 day and 1 episode of blood in the stools. There was history of increased irritability, poor feeding and drawing up of the legs during the crying episodes. He was lethargic on examination with a heart rate of 152 per minute, blood pressure of 118/76 mmHg, oxygen saturation of 100%, capillary refill of 2–3 s and respiratory rate of 40 per minute. On examination, the abdomen was distended with an ill-defined mass palpable in the left hypochondrium. He had another episode of diarrhea while in the ED which also was mixed blood and mucous. The initial venous blood gas showed a pH of 7.424, PCO_2_ 36 mmHg, HCO_3_ 23 mmol/L, base excess −0.6 mmol/L, Na 132 mmol/L, K 3.7 mmol/L, iCa 1.21 mmol/L, glucose 7.7 mmol/L and lactate 1.40 mmol/L. The patient was kept nil by mouth and started on maintenance intravenous fluids. A pediatric emergency physician specialized in POCUS performed an abdominal POCUS using with the low-frequency transducer (5–2 MHz) and linear transducer (8–5 MHz). This showed the presence of doughnut or target sign on the axial sonography in the left upper quadrant (Fig. 1). Detailed sonological assessment of the area revealed sub-serosal echogenic foci in the intestinal wall. This sub-serosal echogenic foci was suggestive of sonographic pneumatosis intestinalis or intramural air (Fig. 1). Abdominal X-rays done showed gasless abdominal field except in the gastric area (Fig. 2).

Comprehensive ultrasound was done by the radiologist showed ileo-colic intussusception with marked thickening of the bowel wall. Vascularity was not demonstrated on the Doppler and an echogenic foci of intramural air was also noted. A trial of pneumatic reduction was attempted which was unsuccessful. The patient then underwent laparotomy which revealed ischemic terminal ileum and caecum. A right hemicolectomy with reduction of intussusception and anastomosis of bowel was done. Histopathological evaluation revealed edematous and dusky bowel in the resected specimen. Post-operative period was uneventful and he was discharged after 5 days.

## Discussion

There is a growing interest in using POCUS for diagnostic assistance for intra-abdominal pathologies in ED and critical care settings [13]. In this case, we present the first report of ultrasound application being used by pediatric emergency physicians to detect pneumatosis intestinalis in abdominal pathologies.

The diagnosis of intussusception by POCUS requires the presence of doughnut or target sign on the axial sonography or the pseudo-kidney sign on longitudinal scans [14, 15]. Ultrasound depiction of pneumatosis intestinalis in bowel wall in the form of echogenic foci or lines is a predictor of non-reducibility during air enema with a sensitivity of 47% and specificity of 96% [5]. The presence of pneumatosis intestinalis in intussusception indicates bowel ischemia [5]. The concurrent presence of sub-serosal air significantly decreased the chances of successful reduction of intussusception [5]. In our patient, the plain film done to detect pneumatosis intestinalis was negative for intramural air but the POCUS had detected the presence of intramural air. Pneumatosis intestinalis appears as echogenic dots and lines in the bowel wall and can be easily detected in the edematous, thickened bowel [5]. The detection of sub-serosal air is relatively difficult because of the presence of concurrent intraluminal air. Hence, the presence of the intramural and sub-serosal air should be confirmed on both long-axis and short-axis views of the bowel. The histopathologic diagnosis of pneumatosis intestinal can be made on the resection specimens by the presence of submucosal or sub-serosal empty spaces which is lined by histiocytes and giant cells [16].

The POCUS of the abdomen to detect the features of small and large bowel pathologies must be done with the low-frequency transducer (5–2 MHz) to begin with to get a general overview of the abdomen. This must be followed up by a systematic scanning of the bowel using the linear transducer (8–5 MHz). Use of adjuncts such as color and power Doppler can give vital information about the blood flow in the intestinal wall. The large bowel assessment is started at the right lower quadrant then proceeding upwards along the length of the ascending colon. This is then continued to the upper abdomen along the length of transverse colon and followed by the descending colon till the left lower quadrant [17]. To assess the morphology of the small bowel, the scanning must be done in the central region of the abdomen. The technique of graded compression should be used to obtain an adequate view of the small bowel by gently displacing the gas or liquid material present within the intestinal lumen [18]. The presence of intramural air must be confirmed in both short and long axis of the bowel.

The detection of pneumatosis intestinalis by POCUS done by the emergency medicine physician can alert the treating team towards the presence of an advanced stage of intussusception and bowel ischemia. These cohorts of patients’ need careful risk stratification and the decision regarding the need for surgical intervention has to be taken at the earliest [5]. It was noted by the authors that plain abdominal radiographs (supine and left lateral decubitus) in this case study did not reveal X-Ray features of pneumatosis despite earlier detection by point-of-care ultrasound. The presence of pneumatosis intestinalis may help indicate severity in intussusception. Possible ultrasonographic risk stratification for operative reduction and unsuccessful non-surgical reduction for intussusception can be derived based on the presence of intramural air [5]. Sonologic detection of pneumatosis intestinalis in combination with other findings such as bowel wall ischemia (no flow on Doppler), peritoneal fluid and enlarged lymph nodes on the ultrasound may prognosticate failure of non-surgical reduction of intussusception. [14, 19]. Detection of pneumatosis intestinalis has also implications on early detection of enterocolitis especially in premature neonates presenting to the ED with gastrointestinal symptoms.

In this case, the POCUS result was useful in clinical decision making. Better understanding of the role of POCUS in evaluating children’s acute abdominal pain and presence of pneumatosis intestinalis can help avoid radiation exposure for diagnostic purposes in this radio-sensitive population. This case cannot be used to make conclusions about the epidemiology of intramural air and its significance in pediatric gastrointestinal pathologies nor the diagnostic accuracy of POCUS for detecting this condition in children, but suggests that further research into this promising modality is warranted.

## References

[1] Bloom RA, Craciun E, Lebensart PD, Levy P, Ziv JB (1992). The ultrasound appearances of intramural bowel gas: the bright ring appearance and the effervescent bowel. A report of three cases. Br J Radiol.

[2] Oktar SO, Yücel C, Erbaş G, Ozdemir H (2006). Use of twinkling artifact in sonographic detection of intestinal pneumatosis. Abdom Imaging.

[3] Romano S, Lassandro F, Scaglione M, Romano L, Rotondo A, Grassi R (2006). Ischemia and infarction of the small bowel and colon: spectrum of imaging findings. Abdom Imaging.

[4] St Peter SD, Abbas MA, Kelly KA (2003). The spectrum of pneumatosis intestinalis. Arch Surg.

[5] Stranzinger E, DiPietro MA, Yarram S, Khalatbari S, Strouse PJ (2009). Intramural and subserosal echogenic foci on ultrasound in large bowel intussusception. Prognostic indicator for reducibility?. Pediatr Radiol.

[6] West KW, Rescorla FJ, Grosfeld JL, Vane DW (1989). Pneumatosis intestinalis in children beyond the neonatal period. J Pediatr Surg.

[7] Kim JS (2013). Acute abdominal pain in children. Pediatr Gastroenterol Hepatol Nutr.

[8] Marin JR, Abo AM, Arroyo AC, Doniger SJ, Fischer JW, Rempell R (2016). Pediatric emergency medicine point-of-care ultrasound: summary of the evidence. Crit Ultrasound J.

[9] Menaker J, Blumberg S, Wisner DH, Dayan PS, Tunik M, Garcia M (2014). Use of the focused assessment with sonography for trauma (FAST) examination and its impact on abdominal computed tomography use in hemodynamically stable children with blunt torso trauma. J Trauma Acute Care Surg.

[10] Elikashvili I, Tay ET, Tsung JW (2014). The effect of point-of-care ultrasonography on emergency department length of stay and computed tomography utilization in children with suspected appendicitis. Acad Emerg Med.

[11] Sivitz AB, Tejani C, Cohen SG (2013). Evaluation of hypertrophic pyloric stenosis by pediatric emergency physician sonography. Acad Emerg Med.

[12] Riera A, Hsiao AL, Langhan ML, Goodman TR, Chen L (2012). Diagnosis of intussusception by physician novice sonographers in the emergency department. Ann Emerg Med.

[13] Kameda T, Taniguchi N (2016). Overview of point-of-care abdominal ultrasound in emergency and critical care. J Intensive Care.

[14] del-Pozo G, Albillos JC, Tejedor D (1996). Intussusception: US findings with pathologic correlation—the crescent-in-doughnut sign. Radiology.

[15] Swischuk LE, Hayden CK, Boulden T (1985). Intussusception: indications for ultrasonography and an explanation of the doughnut and pseudokidney signs. Pediatr Radiol.

[16] Koreishi A, Lauwers GY, Misdraji J (2007). Pneumatosis intestinalis: a challenging biopsy diagnosis. Am J Surg Pathol.

[17] Lim JH (2000). Ultrasound examination of gastrointestinal tract diseases. J Korean Med Sci.

[18] Worrell JA, Drolshagen LF, Kelly TC, Hunton DW, Durmon GR, Fleischer AC (1990). Graded compression ultrasound in the diagnosis of appendicitis. A comparison of diagnostic criteria. J Ultrasound Med.

[19] Koumanidou C, Vakaki M, Pitsoulakis G, Kakavakis K, Mirilas P (2002). Sonographic detection of lymph nodes in the intussusception of infants and young children: clinical evaluation and hydrostatic reduction. AJR Am J Roentgenol.

